# Is there an association between diabetes and neck and back pain? An updated systematic review with meta-analyses

**DOI:** 10.1186/s12998-026-00650-2

**Published:** 2026-05-21

**Authors:** Hannah Dale, Daniel Pozzobon, Matthew Fernandez, Rhys Dale, Isabelle Scott, Shadi Khadijeh Gholami, Eric L. Hurwitz, Katie de Luca

**Affiliations:** 1 School of Health, Medical and Applied Sciences, CQUniversity, Brisbane, QLD 40001 Australia; 2 Institute of Bone and Joint Research, Kolling Institute, Sydney Medical School, University of Sydney, Sydney, NSW Australia; 3 Private Practice, Brisbane, Australia; 4 International Medical University, Kuala Lumpur, Malaysia; 5 Epidemiology Program, Department of Public Health Studies, Thompson School of Social Work and Public Health, University of Hawai’i, Mānoa, Honolulu, Hawai’i USA

**Keywords:** Diabetes, Back pain, Neck pain, Spine pain

## Abstract

**Background:**

Spinal pain and disorders are a major burden on our population and health systems. Previous research showed people with diabetes were 1.35 times and 1.24 times more likely to report low back pain and neck pain than people without diabetes respectively, but diabetes was not a predictor of future spinal pain. Here we update a previous systematic review to determine whether in an adult population, diabetes is associated with low back, neck, or spinal pain.

**Methods:**

The search was performed in PUBMED, Medline, CINAHL and EMBASE. Studies between 2017–2025, of cross-sectional, case–control, twin-control, and cohort designs, assessing diabetes and low back, neck, or spinal pain in adult populations, were included. Excluded were single-case studies, spinal pathologies, multiple chronic diseases, additional pain sites, and gestational or pre-diabetes. Two independent reviewers extracted data on the incidence of pain and reported associations. Odds ratios (ORs) of the association between the exposure (diabetes) and outcome (back, neck, or spinal pain) were calculated using a random effects model. Systematic review registration: PROSPERO registration CRD42022333064.

**Results:**

A total of 15 studies were included with 11 studies included in the meta-analysis. When compared to people without diabetes, people with diabetes had 1.44 times higher odds of reporting low back pain (n = 592,901; OR 1.44; 95% Confidence Interval (CI) 1.20- 1.74; *p* = 0.001), a 1.11 times higher odds of reporting neck pain (n = 465,130; OR 1.11; 95%CI 1.13–1.20; *p* =  < 0.001) and a 1.42 times higher odds of reporting spinal pain (n = 1,335,999; OR 1.42; 95%CI 1.26–1.63; *p* =  < 0.001). Diabetes was significantly associated with 1.27 times higher odds of developing spinal pain (n = 35,435; OR 1.27; 95%CI 1.08–1.50; *p* = 0.004) and 1.44 higher odds of developing neck pain (n = 15,075; OR 1.44; 95%CI 1.09 -1.90; *p* = 0.010). There was no significant association between diabetes and developing low back pain (n = 21,215; OR 1.11; 95%CI 0.88–1.41, *p* = 0.388).

**Conclusions:**

With the inclusion of fifteen additional studies providing an overall sample size five and a half times larger than the previous review, diabetes was associated with prevalent neck, low back and spinal pain, as well as incident neck and neck and spinal pain. Clinicians must be adept at identifying co-morbid conditions and risk factors, understanding the interplay between them and creating well managed networks to address these complex presentations.

**Supplementary Information:**

The online version contains supplementary material available at 10.1186/s12998-026-00650-2.

## Background

Spinal pain, disorders, and spinal pathologies are increasingly common in our society and represent a major burden on our population and health system [1]. The 2020 Global Burden of Disease Study found that 619 million people, almost 10% of the global population, suffered from low back pain. This figure is projected to rise to 843 million by 2050 [2]. Neck pain affected 203 million people in 2020, expected to reach 269 million by 2050 [3]. The study also found that back and neck pain was the leading cause of years lived with disability (YLDs) and the sixth leading cause of disability-adjusted life year (DALY) [4, 5].

In 2016 the Global Spine Care Initiative (GSCI) conducted a scoping review and found a range of co-morbidities were associated with spinal pain [6]. The most frequently associated health conditions were musculoskeletal pain, diabetes mellitus and headaches. The scoping review was unable to provide a clear indication as to which conditions were modifiable risk factors, and which were co-morbidities and the authors highlighted the need for further high-quality research to clarify the relationship between diabetes and spinal pain, with the aim of reducing the burden of common spinal disorders on overall health and wellbeing [6].

Diabetes mellitus Types 1 & 2 (DM) are a metabolic disease, that chronically impairs the body’s ability to produce or counter insulin and leads to compromised carbohydrate metabolism and elevated levels of blood glucose [7, 8]. In 2019 the global prevalence of diabetes mellitus was estimated to be 463 million and expected to rise to 578 million by 2030 [9]. Several factors may contribute to the association between diabetes and spinal pain, such as pathological changes in spine structure, loss of disc height, decreased vertebral bone mass, increased pain-sensitive structures around the lumbar vertebrae, and endplate sclerosis [10] as well as nerve damage, costochondritis, obesity, decreased muscle strength, depression, and an inactive lifestyle. People with diabetes experience changes in the blood flow at a micro and macro level [7] which can lead to impaired function and repair of muscles, ligaments and discs [10, 11]. Coupled with increased inflammation, this can predispose patients to disc degeneration, spinal stenosis, and associated spinal pain [12–14]. Chronic reduction of blood flow and repair is also a mechanism that can lead to muscle wastage, found in people with diabetes with and without polyneuropathy [15]. As muscles, tendons and ligamentous structures degenerate, stability is lost leading to greater load on the spine [16]. Muscle atrophy is compounded, in many cases by a sedentary lifestyle, a risk factor in both the spinal pain and diabetes groups [16]. Conversely, exercise has been shown to improve lower back pain [17] and diabetes [18].

A 2019 meta- analysis of eleven cross-sectional, case–control, twin-control and cohort observational studies evaluated the associations between Type 1 or Type 2 diabetes and non-specific back, neck or spinal pain [14]. The review found people with diabetes were 1.35 times more likely to report low back pain and 1.24 times more likely to report neck pain compared to those without diabetes. Notably, this significant association was found to be independent of BMI. In the review, one longitudinal cohort study (n = 1,284) [19] was included, that suggested diabetes was not a predictor for, or associated with, future risk of developing spinal pain, however the authors noted that the follow up time may have been insufficient to demonstrate an association. Since 2019, several new large scale and longitudinal studies of interest have been conducted, necessitating an update of the review. Therefore, the aim of this systematic review was to update the previous systematic review undertaken by Pozzobon et al. [14], and determine whether in an adult population, diabetes is associated with spinal pain.

## Methods

### Study design

This systematic review was completed using PRISMA guidelines (Supplementary material 1) and prospectively registered on PROSPERO on 23rd June 2022 (CRD42022333064; https://www.crd.york.ac.uk/prospero/display_record.php?RecordID=333064).

### Search strategy

#### Data sources and searches

The search strategy used the following electronic databases: PUBMED, Medline, CINAHL and EMBASE, and studies between 2017 and 2025 were included. The keywords used in the search were based on the exposure (diabetes), outcome (neck pain, back pain, spinal pain), and study design (e.g. observational studies such as cohort and cross-sectional studies); (Supplementary material 2). Inclusion criteria were study design (cross sectional, case control, twin-control, and cohort studies); adults aged 18 years and above, exposed and unexposed to diabetes type 1 and type 2 (self-reported and/or medically diagnosed); and reported low back, neck and spinal pain. Only full text articles were included and published in English. Animal studies, randomized control trials (RCTs) and single case studies were excluded, and population samples with spinal pathology (i.e. cancers, bone disease), additional pain sites, and multiple chronic diseases were excluded. Studies that included gestational and pre-diabetes were also excluded.

### Study selection

Citations identified from databases were imported to EndNote and after removing duplicates, two independent reviewers (HD and RD) performed title and abstract screening. Any disagreements were discussed and resolved by a third reviewer (KD). Two reviewers (HD and RD) then independently screened the remaining abstracts using the same procedure, and potential full texts were then retrieved and screened using the same procedure.

### Data extraction and management

Two independent reviewers (HD and RD) used a standardised data extraction form to extract data related to study design, sample characteristics, back pain, neck pain and spinal pain diagnosis and symptoms, diabetes, and quantitative study results. A third author (KD) resolved disagreements in data extraction if required. Where data were not available, study authors were contacted to request additional information. Studies were grouped based on site of pain and study design.

### Assessment of methodological quality

The methodological quality of the included studies was assessed by two independent reviewers (HD and IS) using the Newcastle–Ottawa Scale (NOS) as recommended by the Cochrane Collaboration [20]. The NOS scale is used to assess case–control and cohort studies, and this review includes a modified version of the NOS scale for cross-sectional studies based on previous studies [21]. The scale assesses for Selection, Comparability and Exposure/Outcome. Studies are graded one point each for all items except comparability which has the potential to score up to two points. The overall quality of the studies were rated from 0–9, with studies rated 0–2 poor quality, 3–5 fair quality, and 6–9 good/high quality [21, 22]. A third author (KD) resolved disagreements in methodological quality assessment.

### Data synthesis

Data from the previous systematic review [14], were included for data synthesis when estimates were presented as odds ratios (ORs) comparing diabetes and neck pain, back pain or spine pain. We calculated ORs for the association between the exposure (diabetes) and the outcome (back pain only and neck pain only analysed separately and combined as spinal pain) and 95% confidence intervals (CIs), using a random effects model. Between-study heterogeneity was calculated using I2 (I2 ≤ 25% small heterogeneity; 25% > I2 < 75% moderate heterogeneity; I2 ≥ 75% large heterogeneity). All meta-analyses were conducted using Comprehensive Meta-Analysis software (Comprehensive Meta-Analysis, Englewood, New Jersey, US.)

## Results

### Search strategy

**Fig. 1:**
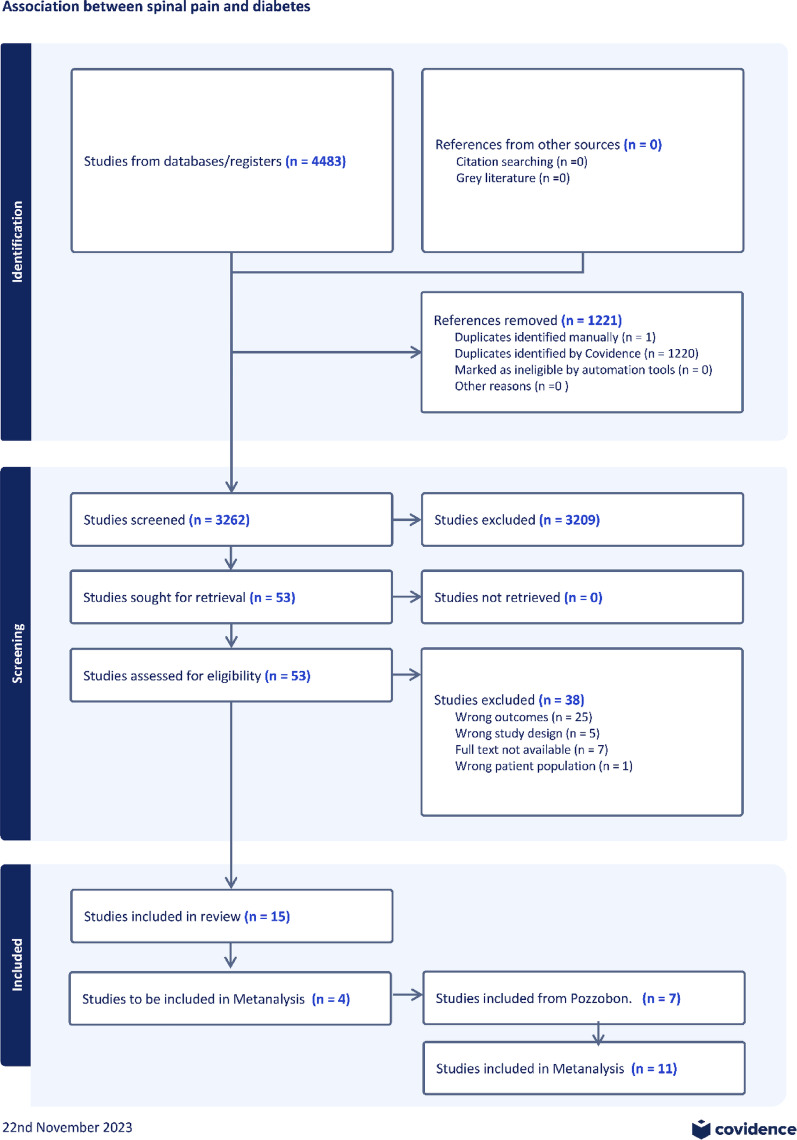
PRISMA flow chart

This search strategy identified 4483 studies. After removing 1221 duplicates and screening titles, 52 full-text studies were assessed for eligibility. There were 38 studies excluded, 25 for wrong outcomes, 5 for wrong study design, 7 did not have a full text not available and 1 study had the wrong patient population. There were 15 studies included for narrative review [23–37]. Please see Fig. 1 for the PRISMA flow chart.

### Included studies

The fifteen studies had a total sample size of 952,643 participants. Data were reported from 13 countries: Saudi Arabia [23], UK [24], Poland [25], Australia [26], Canada [27], Finland [28], Norway [29], Taiwan [30], Germany [31], Spain [32], Brazil [33], Denmark [34, 35] and the USA [36, 37]. Type 2 diabetes was reported on in five studies [24, 25, 28, 31, 33]. Two studies had data separate for Type 1 and Type 2 diabetes [34, 36] and eight studies did not differentiate between Type 1 and Type 2 diabetes [23, 26, 27, 29, 30, 32, 35, 37].

There were nine cross sectional studies [23–25, 27, 28, 33–36] with one study also having longitudinal data [24], four stand-alone longitudinal studies [26, 29, 30, 37], and two case–control studies [31, 32]. Twelve studies reported prevalence data for back or neck pain amongst people with diabetes and those without [23, 25, 26, 28, 29, 32–37] and one study reported bivariate data on the prevalence of diabetes and back pain, neck pain or spinal pain [36]. Odds ratios were reported in four studies on neck pain, back pain and spinal pain in people with diabetes [24, 32, 35, 37]. Hazard ratios for back pain amongst people with diabetes were reported in two studies [30, 31]. Risk ratios for diabetes among back pain patients, and back pain amongst people with diabetes reported in one study [29]. There were eleven studies that adjusted for sex and age as variables in their analyses [23, 24, 26, 29–33, 35–37]. There were five studies that presented stratified data on sex differences [24, 29–32]. Measures of obesity or BMI were adjusted in seven studies [23, 24, 29, 32, 33, 35, 36], while sociodemographic factors were considered in eight studies [23, 24, 26, 30, 33, 35–37]. Various health factors including smoking, physical activity, blood markers, medication and multiple morbidities were also adjusted for in eighteen studies [23, 24, 26, 28–33, 35–37]. Descriptive Tables 1, 2 and 3 were created from the data extracted to describe the studies characteristics.

**Table 1:** Cross-sectional studies

Author, year	Study sample	Assessment of diabetes	Assessment of spinal pain	Results	Quality score
Aldossari 2020	Saudi Arabia Cross-sectional N = 1,019 Participants aged 18–67 years F 628 M 375 Diabetes (Type 1 & 2)	Medically diagnosed	Self-reported neck pain and low back pain	Prevalence of neck pain 4.4% Prevalence of low back pain 8.9% Diabetes N = 45	Fair
Carvalho 2020	United Kingdom Cross -sectional N = 495,327 Mean age (SD) 56.5 (8.1) F 269,755 Diabetes (Type 2) Longitudinal N = 168,75 Mean age (SD) 61.6 (7.0) F 8220 Diabetes (Type 2)	Self-Reported	Self-reported neck pain and low back pain	Prevalence of neck pain OR 1.2, 95% CI, 1.1 -1.2 Prevalence of low back pain 1.0 OR 95% CI, 0.1 -1.1 Diabetes N = 21,889 Incidence of neck pain 1.6 OR, 95% CI, 1.1–2.2 Incidence low back pain 1.1 OR, 95% CI, 0.8—1.6 Diabetes N = 777	Fair
Citko 2018	North-East Poland Cross-sectional N = 609 Mean age 40.1 ± 6.7 years F 362 M 247 Diabetes (Type 2)	Self-Reported	Self-reported low back pain	Prevalence of recurrent low back pain 62.1% Prevalence of chronic low back pain 19.6% Prevalence of recurring low back pain 3.5 OR, 95% CI, 1.2–10.7 Prevalence of chronic low back pain 4.5 OR, 95% CI, 1.1 -17.9 Diabetes N = 58	Fair
Hashem 2018	Canada Cross-sectional N = 76 Mean age 55.9 ± 14.0 M 49.0% Diabetes	Medically reviewed	Medically reviewed low back pain	Prevalence of chronic nonspecific low back pain 10.2% Prevalence of specific low back pain 18.5% Diabetes N = 76	High
Heikkala 2022	Finland Cross-sectional N = 3,478 Mean age (SD) 70.0 (11.7) M 1,899 Diabetes (Type2)	Medically diagnosed	Medically diagnosed neck and low back pain	Prevalence of neck pain 7% Prevalence of low back pain 39% Diabetes N = 753	High
Liaghat 2023	Denmark Cross-sectional N = 3,767 Diabetes (Type 2)	Medically diagnosed	Self-reported cervical, thoracic, and lumbar pain	Prevalence of spinal pain18.5–49.6% Diabetes N = 3,767	Fair
Mendonça 2020	Brazil Cross-sectional N = 150 Mean age 39.6 ± 0.7 F 128 M 22 Diabetes (Type 2)	Medically diagnosed	Self-reported neck pain and low back pain	Prevalence of neck pain 23.3% Prevalence of low back pain 48.3% Diabetes N = 60	High
Rehling 2019	Denmark Cross-sectional N = 109,218 Mean age 65.6 ± 11.0 Diabetes (Types 1 & 2)	Self-reported	Self-reported neck pain and low back pain	Prevalence of neck pain 56.0% Prevalence of low back pain 60.6% Prevalence of neck pain 1.2 OR, 95% CI, 1.1–1.3 Prevalence of low back pain 1.2 OR, 95% CI, 1.1–1.2 Diabetes N = 9,238	High
Rinaldo 2017	United States of America Cross-sectional N = 67,132 Mean age 63.2 (14.8) F 29,225 M 37,907 Diabetes (Types 1 & 2)	Medically recorded	Medically recorded low back pain	Prevalence of low back pain 15% Diabetes N = 9,137	High

**Table 2:** Cohort studies

Author, year	Study sample	Assessment of diabetes	Assessment of spinal pain	Results	Quality score
Carvalho 2020	United Kingdom Cross-sectional N = 495,327 Mean age (SD) 56.5 F 269,755 With diabetes (Type 2) Cohort Longitudinal N = 16,875 10 year follow up Mean age (SD) 61.6 F 8,220 Diabetes (Type 2)	Self-reported	Self-reported neck pain & low back pain	Prevalence of neck pain 1.2 OR, 95% CI, 1.1 -1.2 Prevalence of low back pain 1.0 OR 95% CI, 0.1 -1.1 Diabetes N = 21,889 Incidence of neck pain 1.6 OR, 95% CI, 1.1–2.2 Incidence of low back pain 1.1 OR, 95% CI, 0.8—1.6 Diabetes N = 777	High
Heuch 2019	Norway Cohort N = 45,157 Aged 30–69 years F 23,578 M 21,579 Diabetes (Type 1 & 2) Longitudinal N = 25,774 11 year follow up Diabetes (Type 1 & 2)	Self-reported	Self-reported low back pain	Prevalence of low back pain Females 19.8% 1.0, RR, 95% CI, 0.7–1.5 Males 19.4% 1.4 RR, 95% CI, 1.0 -2.0 Diabetes N = 276 Incidence of low back pain Females 66.62% 1.1 RR, 95% CI, 0.9 -1.3 Males 52.0% 1.1 RR, 95% CI, 0.8—1.4 Diabetes N = 118	High
Hussain 2017	Australia Cohort Longitudinal N = 5058 14 year follow up Mean age 49.2 ± (10.9) F 2479 Diabetes (Type 1&2)	Self-reported	Self-reported low back pain	Incidence of low back pain 82% Diabetes N = 5058	Fair
Suri 2018	United States of America Cohort Longitudinal N = 3,045 11 year follow up Mean age 50 M 3045 Diabetes (Type 1 & 2)	Self-reported	Self-reported low back pain	Incidence of low back pain 6% 1.2 OR, 95% CI, 0.8–1.9 Diabetes N = 112	Fair
Wang 2021	Taiwan Cohort Longitudinal N = 10,470 13 year follow up Diabetes (Type 1 & 2)	Medically recorded	Medically recorded low back pain	Incidence of low back pain 37.6% 1.6 HR, 95% CI, 1.2–2.0 Diabetes N = 173	High

**Table 3:** Case–control studies

Author, year	Study sample	Assessment of diabetes	Assessment of spinal pain	Results	Quality score
Jacob 2021	Germany Case–control Longitudinal N = 139,002 Mean age 62.5 (13.4) F 52% M 48% Diabetes (Type 2)	Medically diagnosed	Self-reported low back pain	Incidence of Chronic low back pain 1.2 HR, 95% CI, 1.1 to 1.4 Diabetes N = 69,501	High
Jimenez-Garcia 2018	Spain Case–control Longitudinal N = 45,030 Age 40 years + F = 53.6% Diabetes (Type 1 & 2)	Self-reported	Self-reported, medically reviewed neck pain and lower back pain	Incidence of neck pain 32.2% 1.2 OR, 95% CI, 1.0–1.4 Incidence of low back pain 37.1% 1.2 OR, 95% CI, 1.1–1.4 Diabetes N = 3,441	High

### Narrative synthesis

Across 15 included studies, a consistent narrative emerges suggesting an association between diabetes and spinal pain, despite variation in study design, population characteristics, and outcome definitions. All cross-sectional studies reported higher odds of neck pain, back pain, or spinal pain among individuals with diabetes compared with those without [23–25, 35]. In cross-sectional studies [27, 28, 33–36], the prevalence of neck pain among people with diabetes ranged from 4 to 56% and the prevalence of low back pain among people with diabetes ranged from 10 to 60%. In cohort studies three studies trended towards association- through different forms of statistical analysis, odds ratio [24] risk ratio [29] and hazard ratio [30]. One study did not find an association [37]. Incidence of low back pain was between 6 and 66% [26, 29, 37]. Both case control studies trended towards association, reporting hazard ratio [31] and odds ratio [32].

### Methodological quality

**Fig. 2:**
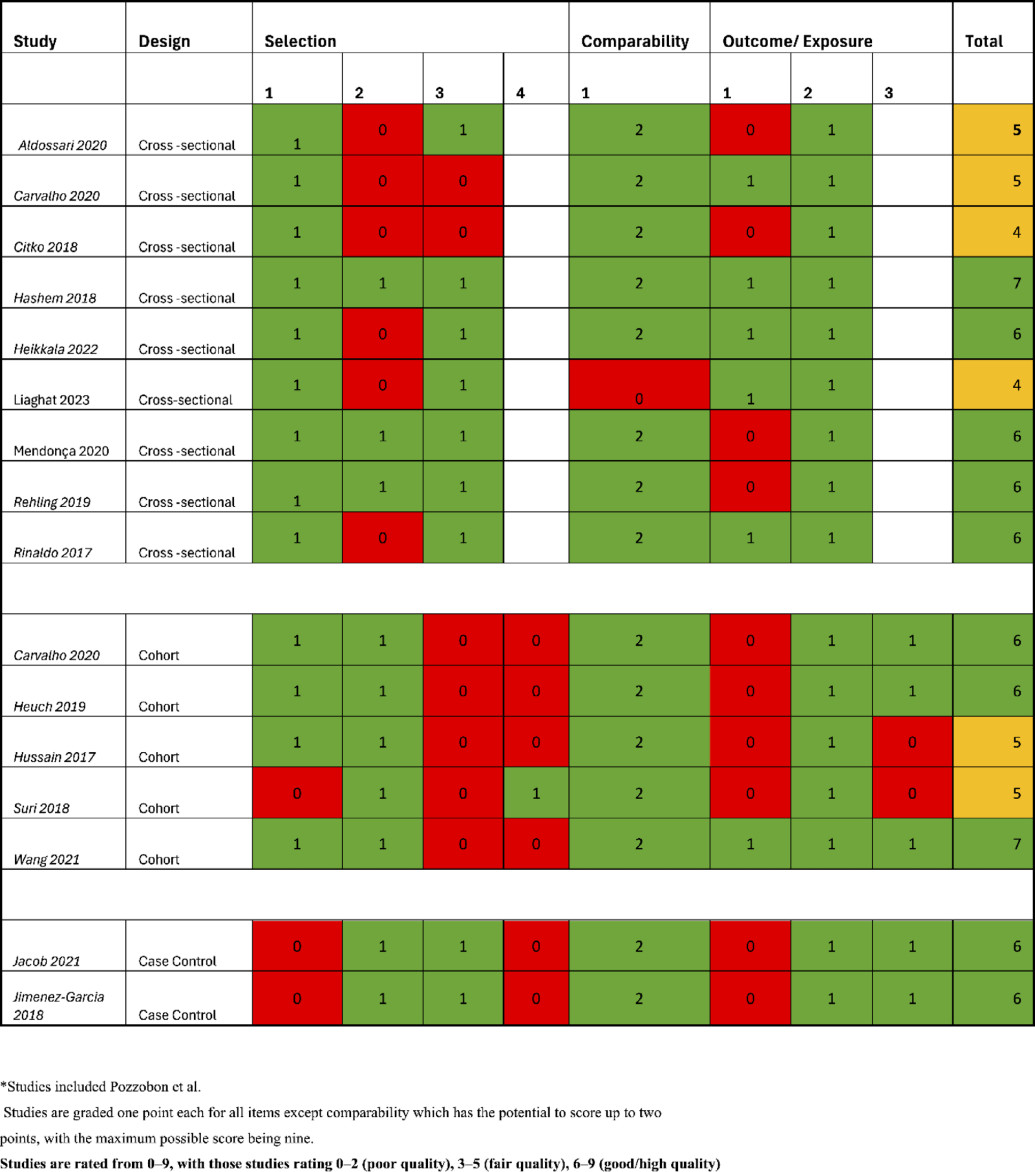
Quality assessment table

Of the fifteen studies, ten were rated as having good/high methodological quality [24, 27–29, 31–33, 35, 36], six studies were rated as fair quality [23–26, 34, 37]. One study was assessed twice due to mixed methodology (cohort and cross-sectional) [24]. A summary of the assessment of methodological quality can be found in Fig. 2. For seven of the included studies [19, 38–43], we utilized the methodological quality scores from the previous review by Pozzobon et al. [14]; these are also displayed in Fig. 2. In regard to the assessment of publication bias, there was no evidence of small study bias was observed for the studies included in our pooled analyses of low back pain, longitudinal low back pain, neck pain or longitudinal spinal pain. In addition to visual inspection of the funnel plots, the results of Egger’s tests also suggest lack of appreciable evidence of publication bias. There was evidence of bias in the visual inspection of the spinal pain funnel plot, which should be considered when interpreting results (Supplementary material 3).

### Meta analysis

#### Association between diabetes and low back pain

**Fig. 3:**
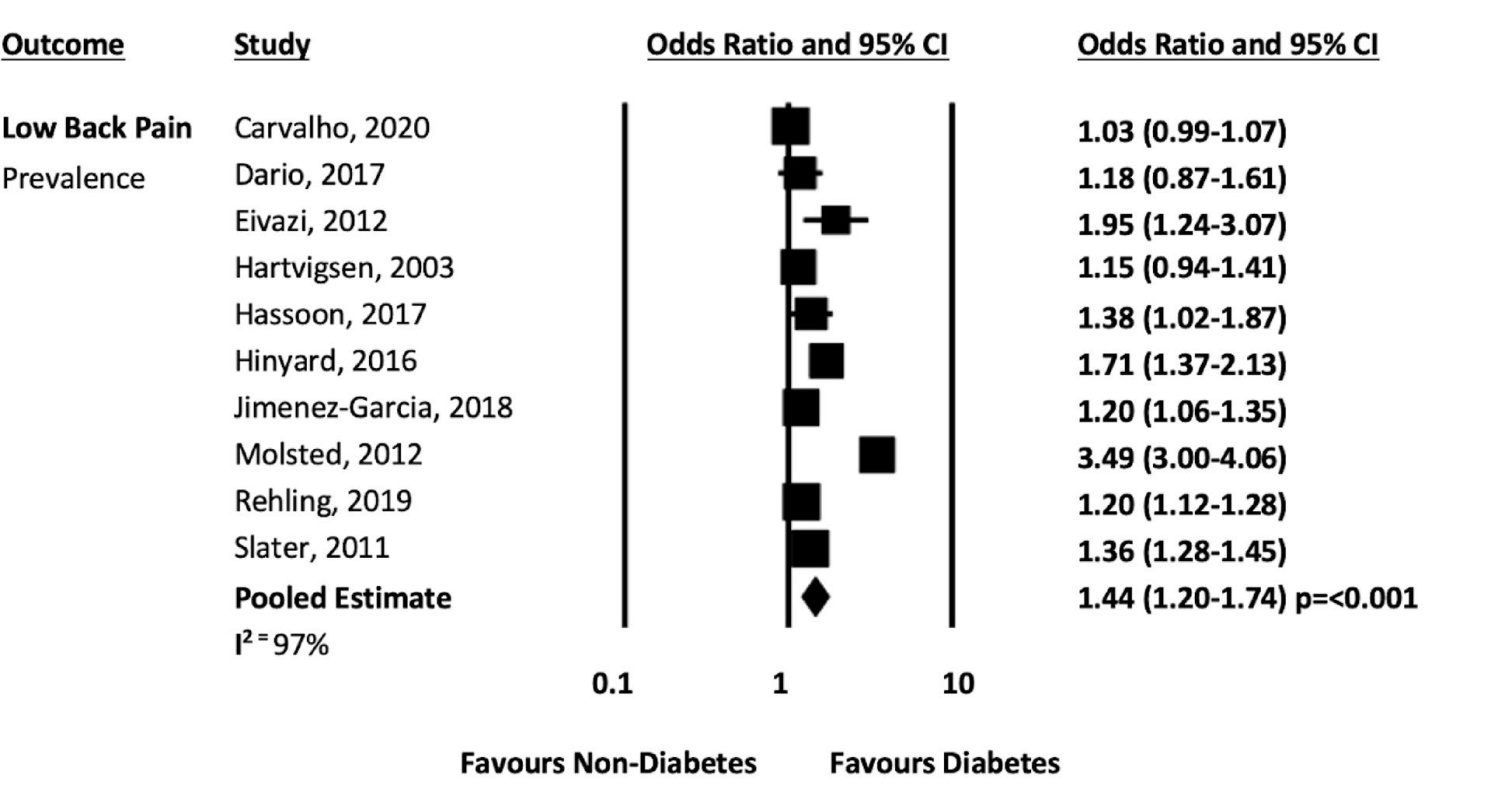
Low back pain prevalence forest plot

**Fig. 4:**
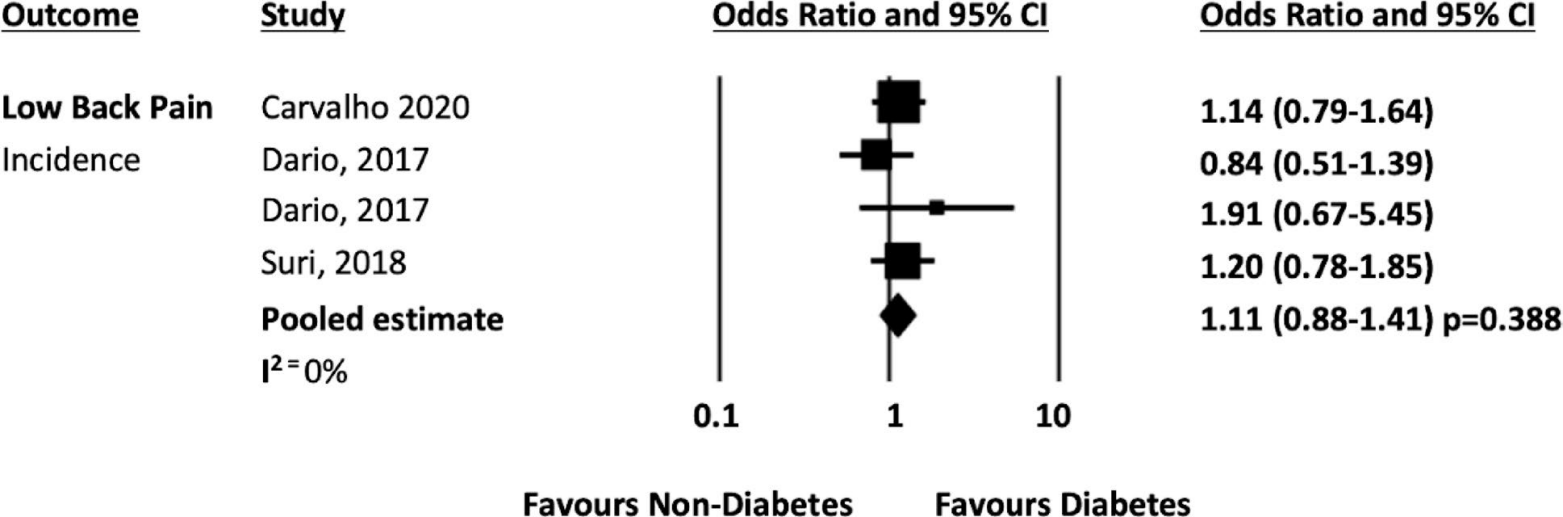
Low back pain incidence forest plot

When combined with data from the previous systematic review [14], 11 studies [19, 24, 32, 35, 37–43] presented data on diabetes and low back pain that could be pooled. The pooled analysis from ten cross sectional studies [19, 24, 32, 35, 38–43] showed that people with diabetes had 1.44 times higher odds of reporting low back pain compared to people without diabetes, (n = 592,901; OR 1.44 95% CI 1.20–1.74, *p* = 0.001; I^2^ = 97%, see Fig. 3). The pooled analysis from three longitudinal studies [19, 24, 37] showed that people with diabetes had 1.11 higher odds of developing low back pain compared to people without diabetes (n = 21,215; OR 1.11 95% CI 0.88–1.41, *p* = 0.388; I^2^ = 0%, see Fig. 4), however this association was not statistically significant.

### Association between diabetes and neck pain

**Fig. 5:**
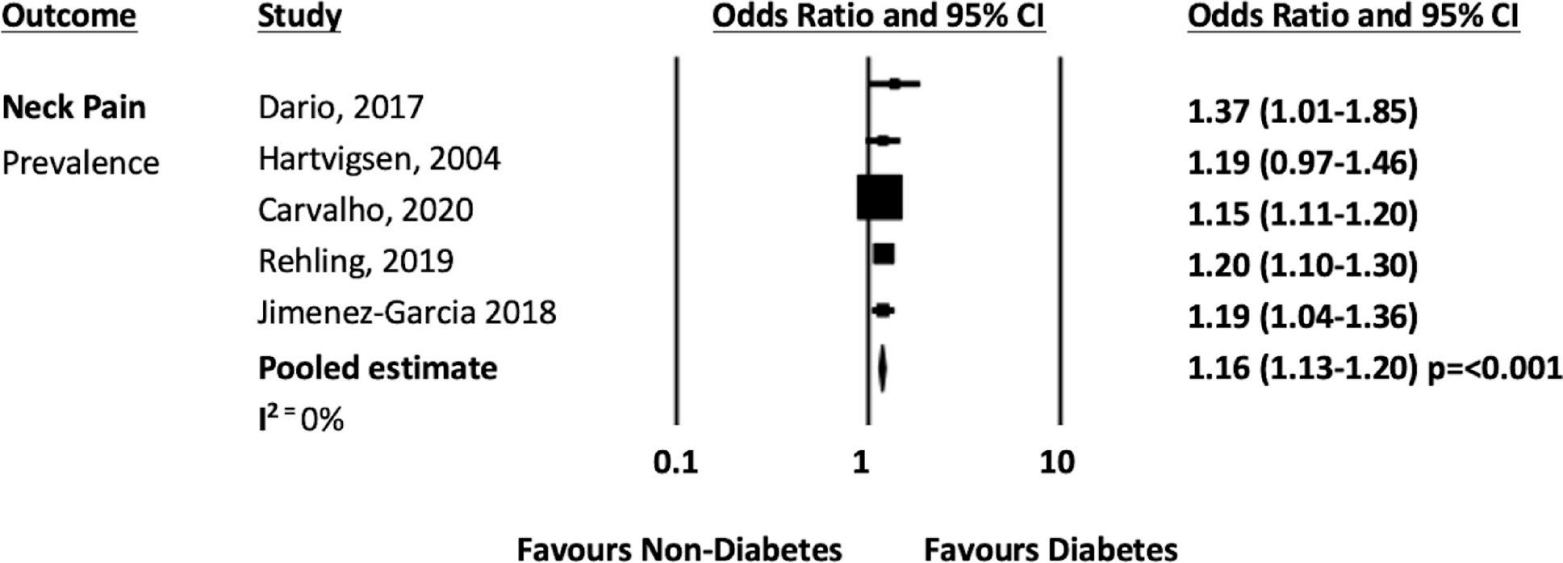
Neck pain prevalence forest plot

**Fig. 6:**
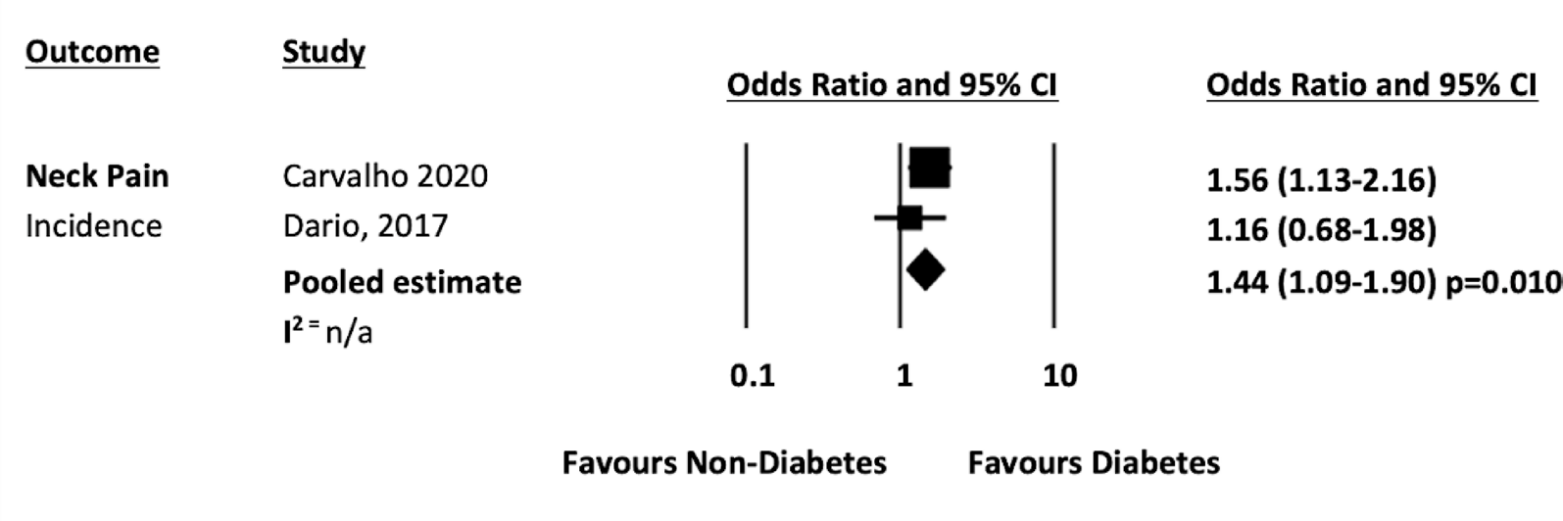
Neck pain incidence forest plot

When combined with data from the previous systematic review [14], five studies [19, 24, 32, 35, 38] presented data on diabetes and neck pain that could be pooled. The pooled analysis from five cross sectional studies [19, 24, 32, 35, 38] showed that people with diabetes was associated with 1.11 higher odds of prevalent neck pain compared to people without diabetes (n = 465,130; OR 1.11 95% CI 1.13–1.20, *p* =  < 0.001; I^2^ = 0%). See Fig. 5). The pooled analysis from two longitudinal studies [19, 24] showed that diabetes had 1.44 higher odds of developing neck pain compared to people without diabetes (n = 15,075; OR 1.44 95% CI 1.09 -1.90, *p* = 0.010; I^2^ = n/a, see Fig. 6).

### Association between diabetes and spinal pain

**Fig. 7:**
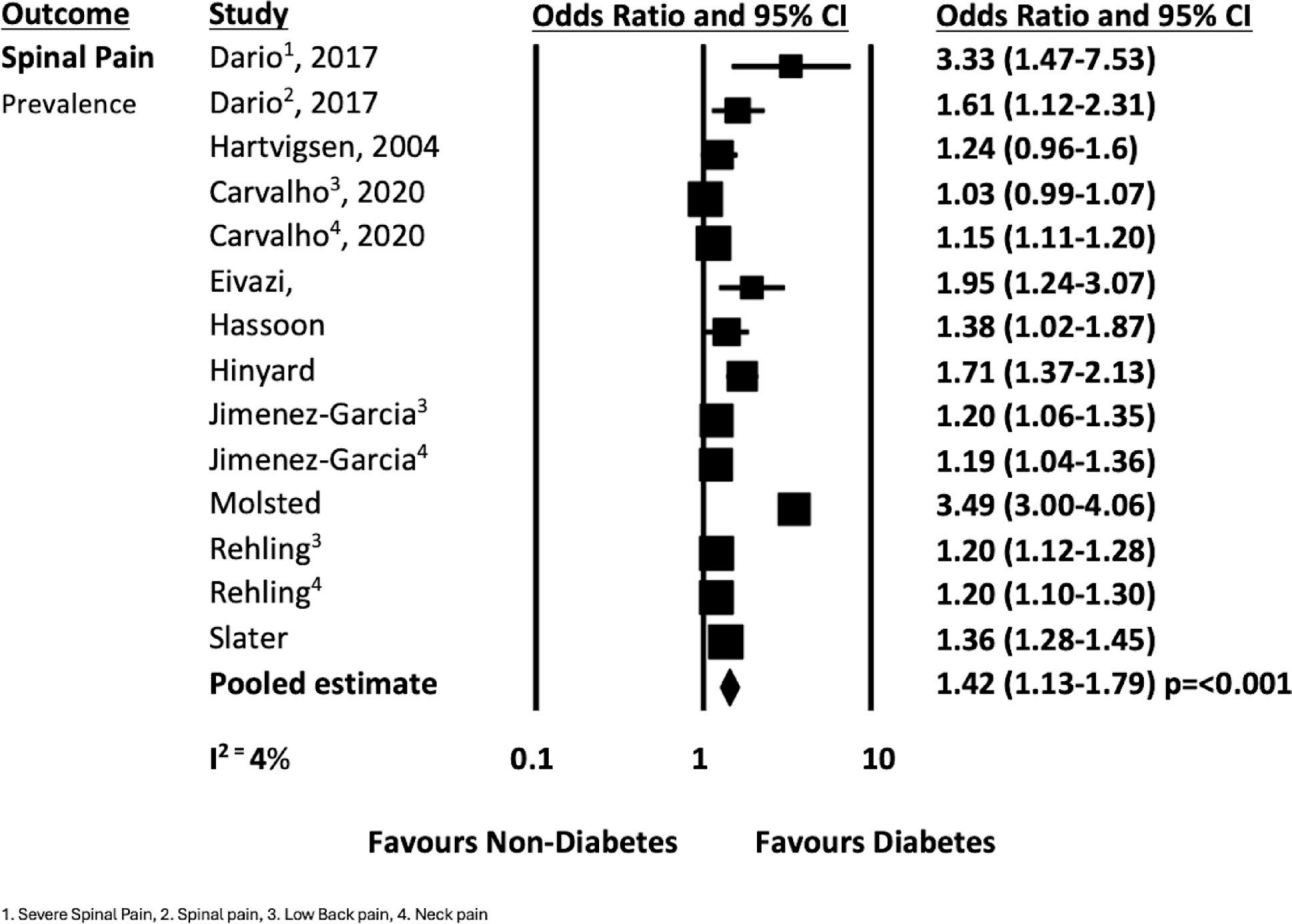
Spinal pain prevalence forest plot

**Fig. 8:**
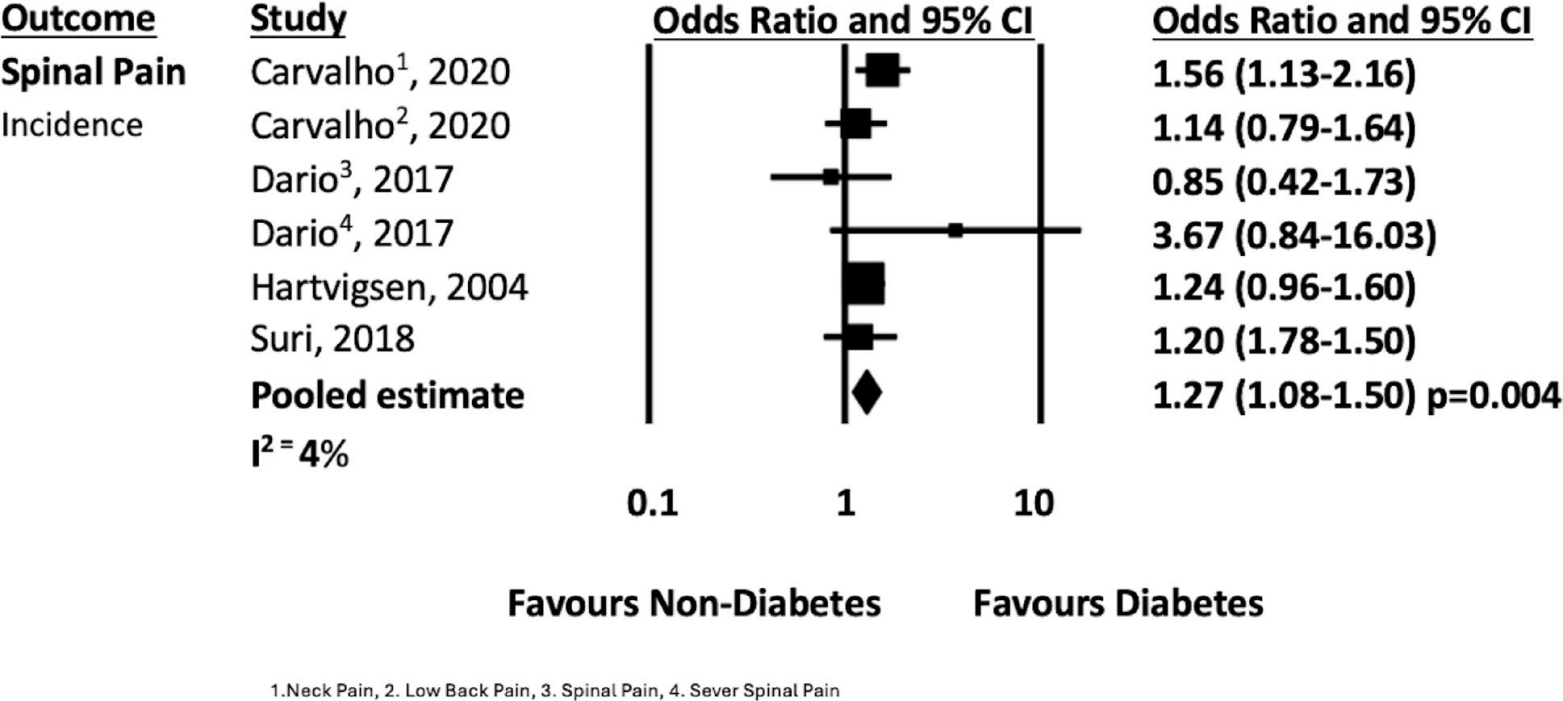
Spinal pain incidence forest plot

When combined with data from the previous systematic review [14], eleven studies presented data on diabetes and spinal pain (analyses of back pain, neck pain and spinal pain) that could be pooled [19, 24, 32, 35, 37–43]. The pooled analysis from ten cross sectional studies [19, 24, 32, 35, 38–43] showed that people with diabetes had 1.42 times higher odds of reporting spinal pain compared to people without diabetes (n = 1,335,999; OR 1.42 95% CI 1.26–1.63, *p* =  < 0.001; I^2^ = 96%, see Fig. 7). Four longitudinal studies [19, 24, 37, 38] showed that people with diabetes had with 1.27 times higher odds of developing spinal pain compared to people without diabetes (n = 35,435; OR 1.27 95% CI 1.08–1.50, *p* = 0.004; I^2^ = 4%, see Fig. 8).

## Discussion

This review aimed to determine whether in an adult population, diabetes is associated with spinal pain by evaluating and synthesising existing literature on the prevalence and incidence of spinal pain, including low back and neck pain, in people with diabetes We updated the previous review by Pozzobon’s et al. with the inclusion of an additional fifteen studies [14] which provides additional insight and updated estimates. In our update, the overall sample size is five and a half times larger, and therefore stronger associations were observed in cross-sectional data, particularly for low back and spinal pain, with higher ORs than previously reported. With an increase in longitudinal data available, we build on previous findings by providing a clearer understanding of associations over time. These results indicate that people with diabetes are at risk of developing neck pain and spinal pain, and a non-significant trend towards developing low back pain as well, compared to those without diabetes.

Three longitudinal studies in our meta-analysis had follow up periods of between 4–12 years, whereas the single longitudinal study within the 2019 review [19] had a 4 year follow up period, thus strengthening the temporality of results. Unfortunately, three longitudinal studies [29–31] were unable to be included meta-analyses due to the non-homogenous presentation of data, specifically, they reported Hazard Ratios (HR) and Risk Ratios (RR), which could not be pooled with the Odds Ratios (OR) used in the other studies. Jacob et al. [31] and Wang et al. [30] both reported positive associations, with hazard ratios of 1.23 (95% CI: 1.13–1.35) and 1.55 (95% CI: 1.20–2.01; *P* = 0.0008) respectively, suggesting an increased risk of low back pain over time. Heuch et al. [29], found that men with a diagnosis of diabetes may have a higher risk of subsequently experiencing chronic low back pain with a relative risk of 1.43 (95% CI 1.04 to 1.96, *p* = 0.043).

Diabetes may contribute to spinal pain through several pathways, including vascular, inflammatory, and degenerative mechanisms, making the association complex and multifactorial. For instance, increased glucose levels in diabetes can saturate the glycolytic pathways and activate other pathways, which eventually increases the level of advanced glycation end products. This increases reactive oxygen species and leads to inflammation [12]. Hyperglycaemia-induced inflammation in the intervertebral disc may promote intervertebral disc degeneration [10]. Chronic hyperglycaemia may damage peripheral nerves, leading to pain, tingling and numbness. Neuropathy can also alter gait movement and enhance the stress on muscles and joints [44]. Hyperglycaemia further affects musculoskeletal tissues by impairing tendon integrity, increasing susceptibility to tendinopathies [4] and impacts healing processes as reported in a mouse model of diabetes and obesity [45]. Micro and macrovascular changes in various tissues [7] which alter tissue perfusion contribute to muscle cramps and pains and impairs function and repair of muscles, ligaments and discs [10, 11]. Muscle wastage may occur with or without polyneuropathy [15] and as muscles, tendons and ligamentous structure degenerate, stability is lost leading to greater load on the spine [16]. The combined effects of inflammation, impaired tissue repair, biomechanical alterations, and obesity can predispose patients to disc degeneration, spinal stenosis and associated spinal pain [12–14]. The increased loads on lumbar discs compared to cervical discs [46] may help explain the higher odds ratio of having diabetes and low back pain than neck pain. A better understanding of physiological mechanisms underlying spinal pain and diabetes may eventually improve clinical practice through supportive best practices for patient centred treatments. This could include supporting interdisciplinary health teams, improving communication across providers, facilitating timely referrals to medical specialists and ensuring appropriate investigations to enable coordinated, comprehensive, and effective management of individuals with diabetes-related spinal pain.

Physical inactivity may be a shared risk factor for both low back pain and diabetes, with regular movement (with dietary modifications) shown to reduce risk for both. The diabetes and subsequent increased risk of lower back and neck pain can possibly be due to metabolic imbalances that affect spinal structures [47]. Physical activity may help to mitigate inflammation, muscle deconditioning and poor glycemic control [47] associated with diabetes. An understanding of the effects of exercise on the vascular, inflammatory, and degenerative pathways, can inform targeted interventions that address both metabolic and biomechanical contributors to spinal pain in diabetic populations.

We found 11 studies that could be included in a meta-analysis, which all consistently adjusted for sex [19, 24, 32, 38–43]. Several papers included in the meta-analysis also presented sex-specific associations. Jimenez- Garcia et al. found female sex was associated with a 1.77 higher probability of reporting neck pain [32]. Wang et al. found female pharmacists had a 1.12-fold higher risk of low back pain development than the male participants [30], Carvalho et al. stratified analyses found that only women with Type 2 diabetes was associated with recent low back pain and chronic low back pain [24]. The hazard ratio for developing low back pain over 10 years was 1.68 in women, compared to 0.83 in men [31]. Only Heuch et al., found men with diabetes may have a higher risk of chronic low back pain, but made comment that their results need to be confirmed in studies from other populations [29]. Women experience higher rates of low back pain generally compared to men, possibly due to anatomical, hormonal and biopsychosocial factors [48], and within the female population, post-menopausal women experience higher rates than pre-menopausal women. One explanation may relate to the loss of protection effect of estrogen post menopause [49, 50]. Sex-specific associations were not consistent or well documented across the included studies and sex-stratified research is recommended in the future.

### Strengths and limitations

A strength of this systematic review is the inclusion of studies with larger data sets and longer follow up periods, that was up to 14 years. Datasets range from 76 to 495,327 people, collectively close to a million people in 13 different countries, thus enhancing the applicability of our findings across broader populations. Ninety-one percent of the studies adjusted for age or sex, further enhancing the generalisability of the findings. The methodological quality of the studies was predominantly high (65%). We also consulted with CQU librarian to develop a well-defined search strategy, provided a transparent inclusion criteria and conducted a comprehensive literature search on databases, ensuring comprehensive coverage of relevant and available evidence. However, a lack of homogenous data was the major limitation of this review. Eight studies used medical records or a diagnosis of diabetes during the study period to define diabetes [23, 27, 28, 30, 31, 33, 34, 36], while the remaining studies used self-reported data [24–26, 29, 32, 35, 37]. Back pain was mostly self-reported [23–26, 29, 31, 33–35, 37]; two studies used the World Health Organization’s International Classification of Disease [28, 36], and one study used a physician reported definition of low back pain based upon “history, physical exam, and review of cross-sectional imaging”[27]. The reliance on self-reported measures, common amongst observational studies, may limit the precision of exposure and outcome assessment [51]. In cohort studies comparability between respondents and non-respondents’ characteristics was not established in six of the studies [23–25, 28, 34, 36]. These issues represented the main source of low methodological scoring and should be considered when interpreting the results. Furthermore, there was a lack of consistency when collecting and reporting data on either Type 1 or Type 2 diabetes. No studies reported on Type 1 diabetes exclusively, and five studies reported on Type 2 exclusively [24, 25, 28, 31, 33]. Liaghat et al. [34] and Rinaldo et al. [36] reportedly separated data on both Type 1 and Type 2 diabetes, and the remaining eight studies either combined or did not differentiate between Type 1 and Type 2 diabetes [23, 26, 27, 29, 30, 32, 35, 37]. Due to the difference in aetiology, pathophysiology, and treatment approaches, combining data on Type 1 and Type 2 diabetes will impact the understanding of how these factors may relate to, impact or be impacted by comorbidities such as spine pain [48]. It is imperative that future research systematically separates Type 1 and 2 diabetes, to allow for more accurate estimates on the association with spinal pain.

### Future directions

Our new findings strengthen the case for diabetes being a meaningful contributor to neck, back and spinal pain, thus highlighting the need for longitudinal, mechanistic, and interventional research. Specifically, no additional longitudinal studies examining diabetes and future spinal pain have been published and met the inclusion criteria since 2019. Due to the established association between diabetes and current back, neck and spine pain, clinicians require a multidisciplinary approach to spinal care while managing long-term progressions of multimorbidity. The prevalence of chronic low back pain progressively increases from the third decade of life [52], and is one of the major disabling health conditions after 60 years of age [53]. Older adults are also more likely to report multisite pain with persistent back pain and persistent low back pain is less likely to be resolved [54]. Multimorbidity also increases with age and are increasingly prevalent [55]. de Luca et al., reported among older women with spinal pain, the odds of having spinal pain were 2.5 times higher in women with two comorbidities (95%CI 1.47–4.04), and 5.1 times higher in women with four comorbidities (95%CI 1.64–15.54) [56]. While theories for these mechanisms exist, including neuropathy, inflammation, and musculoskeletal changes, further research is needed to establish causal links [10–13, 15, 57]. Obesity, for example, has many similar physiological changes; low-grade systemic inflammation [58], dyslipidaemia [59] and increased biomechanical load [60] and has also been associated with an increased risk of low back pain [61, 62]. In this systematic review, a measure of obesity or BMI was adjusted in seven studies [23, 24, 29, 32, 33, 35, 36] but was not for all studies included in the meta-analysis, diabetes may be a risk factor independent of obesity.

### Clinical implications

Physical inactivity is a shared and modifiable risk factor and yet one in four adults do not meet physical exercise recommendations [63]. Allied health professionals play an important role in exercise promotion and behaviour change [64], which is not traditionally part of the medical school curriculum [65]. Rehling et al. found for people with diabetes there may be a positive effect of physical activity on reduced musculoskeletal pain [35], and Jimenez et al. found more frequent physical activity was associated with a lower self-reported low back pain [32].

Collaborative care teams are suited to managing diabetes related spinal pain, addressing metabolic control, physical activity and spinal health. The Australian model of Chronic Disease Management plans (CDMP) is a step towards affordable multidisciplinary health care, where general practitioners refer to allied health services (i.e. chiropractic, physiotherapy, podiatry) using government funding [66]. CDMPs reduce hospital utilisation and costs for individuals with heart disease or diabetes, and research has shown CDMPs have an increasing effect with continued participation over time [67]. Importantly, multidisciplinary health teams may also reduce medication use [68–70]. Medications that may be used to treat low back pain such as non-steroidal anti-inflammatory drugs, opioids and corticosteroids can adversely affect kidney functions and glycaemic control [57]. Ramanathan et al. found patients suffering with “three or more comorbid conditions were more likely to be prescribed dexamethasone, other oral steroids, antidepressants and more likely to not be advised against resting in bed” [71].

To minimise the risks associated with some pharmacological treatments, it is particularly important to consider non-pharmacological approaches to spinal pain management in people with diabetes. Additionally, individuals with diabetes appear to be at increased risk of progressing to surgical interventions for chronic low back pain [36], despite guideline recommendations that reserve surgery to a limited number of select cases [72]. The option for conservative manual therapy [70] has been associated with clinically relevant improvement in patients who are otherwise surgical candidates [73].

## Conclusion

With the inclusion of fifteen additional studies and larger sample sizes data this review provides greater level of certainty that people with diabetes have higher odds of reporting low back pain, neck pain, and spinal pain when compared to people without diabetes. Additional longitudinal data found people with diabetes have higher odds of developing neck pain and spinal pain, thus offering a definitive update to the existing literature. Clinicians must be adept at identifying co-morbid conditions and risk factors, understanding the interplay between them and creating well managed networks to address these complex issues.

## Supplementary Information

Below is the link to the electronic supplementary material.

Supplementary Material 1

Supplementary Material 2

Supplementary Material 3

## Data Availability

Data is provided within the manuscript or supplementary information files.
