# Supplementary material for: Is there an association between diabetes and neck and back pain? An updated systematic review with meta-analyses
Source: Chiropr Man Therap. 2026 May 21;34:22. doi: 10.1186/s12998-026-00650-2 (PMC13227674; doi:10.1186/s12998-026-00650-2)
Supplement: Supplementary file 2 — Supplementary Material 2 [file 12998_2026_650_MOESM2_ESM.docx]

**SUPPLEMENTARY MATERIAL 2**

A systematic search was conducted in the PUBMED, Medline, CINAHL and EMBASE electronic databases. Articles titles, keywords and abstracts were searched using the following keywords:

**PUBMED, EMBASE strategies**

**CONCEPT 1**

| **Keywords** | **MeSH** |
| --- | --- |
| adult 18 years | adult |

**CONCEPT 2**

| **Keywords** | **MeSH** |
| --- | --- |
| diabetes mellitus insulin resistance diabetes mellitus, type 1 diabetes mellitus, type 2 diabetes type 1 diabetes  type 2 diabetes type 1 diabetes mellitus type 2 diabetes mellitus adult onset diabetes mellitus | diabetes mellitus insulin resistance diabetes mellitus, type 1 diabetes mellitus, type 2 |

**CONCEPT 3**

| **Keywords** | **MeSH** |
| --- | --- |
| cervical vertebrae neck pain back pain low back pain spinal pain  thoracic spine lumbar spine  ache, neck neck ache lower back pain low back ache back ache ache, low back back pain, lower cervical spine | cervical vertebrae neck pain back pain low back pain |

**Medline, CINAHL strategies**

'cervical vertebrae' OR (‘neck pain’) OR (‘back pain’) OR (‘spinal pain’) OR (‘thoracic spine’) OR (‘lumbar spine’) OR (‘ache, neck’) OR (‘neck ache’) OR (‘lower back pain’) OR (‘low back pain’) OR (‘low back ache’) OR (‘back ache’) OR (‘ache, low back’) OR (‘back pain, lower’) OR (‘cervical spine’) OR 'cervical vertebrae' OR 'neck pain'/mj OR 'back pain'/mj OR 'low back pain'/mj

diabetes mellitus OR (‘insulin resistance’) OR (‘diabetes mellitus type 1’) OR (‘diabetes mellitus type 2’) OR diabetes OR (‘type 1 diabetes’) OR (‘type 2 diabetes’) OR (‘type 1 diabetes mellitus’) OR (‘type 2 diabetes mellitus’) OR (‘adult onset diabetes mellitus’) OR 'adult onset diabetes mellitus'/de OR 'diabetes mellitus type 1'/mj OR 'diabetes mellitus type 2'/mj OR 'insulin resistance'/mj
