# Supplementary material for: Is there an association between diabetes and neck and back pain? An updated systematic review with meta-analyses
Source: Chiropr Man Therap. 2026 May 21;34:22. doi: 10.1186/s12998-026-00650-2 (PMC13227674; doi:10.1186/s12998-026-00650-2)
Supplement: Supplementary file 3 — Supplementary Material 3 [file 12998_2026_650_MOESM3_ESM.docx]

**SUPPLEMENTARY MATERIAL 3**

Low back pain prevalence funnel plot


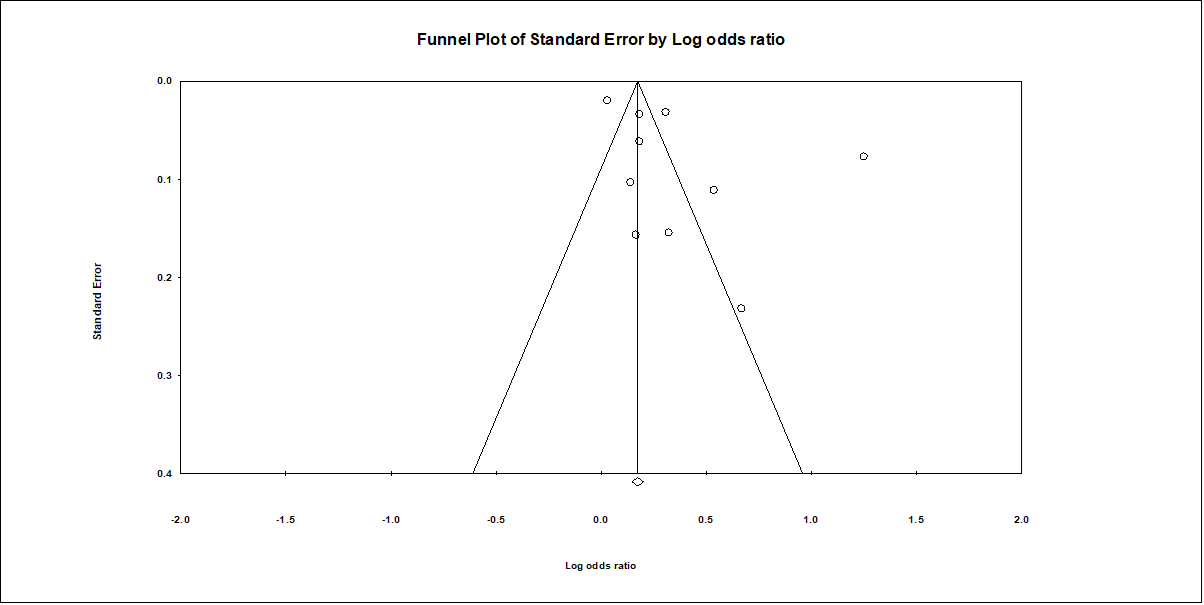


Low back pain incidence funnel plot


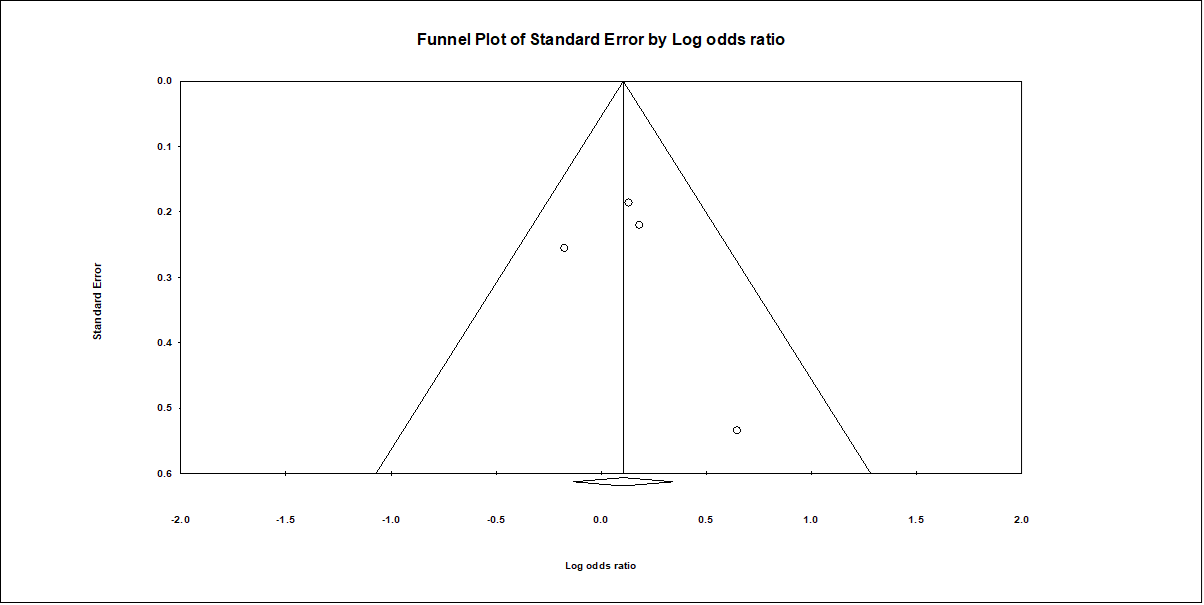


Neck pain prevalence funnel plot


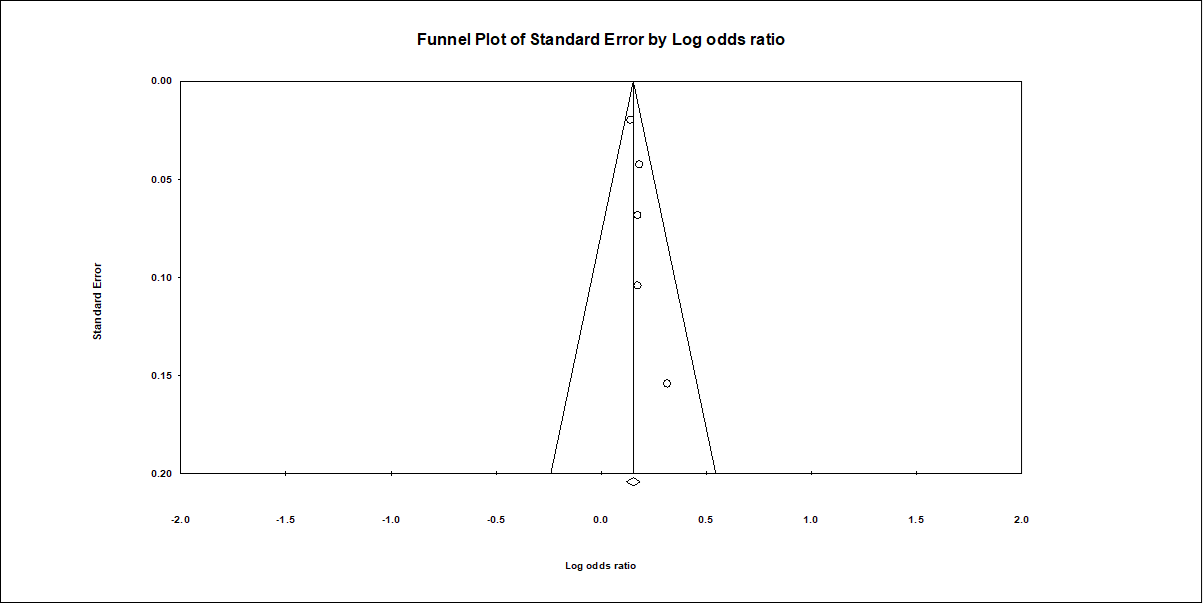


Spinal pain prevalence funnel plot

Spinal pain incidence funnel plot
